# An Auto-Calibrating Semi-Adiabatic Calorimetric Methodology for Strength Prediction and Quality Control of Ordinary and Ultra-High-Performance Concretes

**DOI:** 10.3390/ma15010096

**Published:** 2021-12-23

**Authors:** Marco Viviani, Luca Lanzoni, Vincenzo Savino, Angelo Marcello Tarantino

**Affiliations:** 1 HES-SO/HEIG-VD—Haute École d’Ingénierie et de Gestion du Canton de Vaud, Rte de Cheseaux 1, CH-1401 Yverdon-les-Bains, Switzerland; marco.viviani@heig-vd.ch (M.V.); vincenzo.savino@heig-vd.ch (V.S.); 2 DIEF—Dipartimento di Ingegneria “Enzo Ferrari”, Università di Modena e Reggio Emilia, Via Vignolese 905, 41125 Modena, Italy; angelomarcello.tarantino@unimore.it; 3 CRICT—Centro Interdipartimentale di Ricerca e per i Servizi nel Settore delle Costruzioni, Via P. Vivarelli 10, 41125 Modena, Italy

**Keywords:** maturity, hydration, calorimetry, mechanical properties, concrete, equivalent time

## Abstract

A timely knowledge of concrete and ultra-high-performance concrete (UHPC) strength is possible through the so-called strength-equivalent time (Et) curves. A timely knowledge of concrete strength is useful, for instance, to precisely determine when the shores of a hardening structural element can be safely removed. At the present time, the preparation of the strength-Et curves requires time-consuming and labor-intensive testing prior to the beginning of construction operations. This paper proposes an innovative method to derive the strength-Et and total heat-Et curves for both normal strength and UHPC. Results confirmed that the proposed method is fast, inexpensive, self-calibrating, accurate and can detect any variation of the concrete mix proportions or components quality. In addition, the quality of predictions of strength–maturity curves can be constantly improved as the specimens’ population increases. Finally, results obtained with the proposed method were compared with those obtained using standard methods, showing a good agreement.

## 1. Introduction

In this paper research is presented whose objective is to provide an accurate method to predict the strength of commercial concretes at any time. A second objective of this research is to detect when the mix changes and thus the prediction of the strength is not accurate. The method, composed by a testing apparatus and a model, is conceived to be inexpensive and auto-calibrating, adapted to be used on the field. Predictions of concrete strength are made using the so-called strength–maturity curves. A strength−maturity curve is used to predict the strength of industrial concretes at any time during the hydration process. These curves are constructed in laboratory for a specific mix design. Norms are available to prepare such curves since 1977 and today are mentioned in design norms [1]. The strength values measured in the laboratory are plotted against a *maturity index MI*. *MI* computes the effect of both time and temperature on the *degree of reaction α* of concrete for any curing regime. Cement hydration is faster at high curing temperatures and inversely slower at low curing temperatures. Curing temperatures also influence the hardening properties of the cementitious composite [2]. The laws accounting for the effect of both temperature and time on the hydration speed are not generally linear, and they do not apply to temperatures that are too low or too high. This limitation might be overcome by adopting a different method governed by a resistivity model based on Winner bounds. This method can determine the equivalent time of concrete by measuring the electrical resistivity of concrete, also for wider curing temperature ranges. Nevertheless, very few studies about this method are available [3]. In addition, the method is still not suitable for concrete, due to the poor electrode contact and non-uniformity caused by the presence of coarse aggregate. For this reason, the strength prediction methods based on the maturity indexes are still the most suitable for commercial concretes.

In fact, once a strength–*MI* curve is available for a specific concrete, in order to know the strength at a time t*, it will suffice to monitor the internal temperature history of a hardening structural element made of the same concrete, convert the internal temperature history *T*(*t*) into a *MI*, enter into the strength–*MI* curve the value of *MI* (t = t*) and read the corresponding concrete strength.

Many linear and nonlinear formulas are available to determine *MI* [4,5]. In this paper, the expression maturity is used to indicate whatever formula, linear or nonlinear, used to account for the effect of both time and temperature on the hydration of a cement-based material such as concrete. Freisleben-Hansen and Pedersen *equivalent-time E_t_*(*t*) is a very well-known model used to compute *MI* from the recorded temperature history of concrete and, in turn, to construct the strength–*MI* curves *t* [4,6,7,8,9,10,11,12]. Its significance and limitations have been discussed by the scientific community for decades [13,14,15,16]. The definition of *E_t_*(*t*) is based on the well-known Arrhenius’ law. Such a law is used to determine the rate of a chemical reaction [17]. The time history of a concrete property can be converted into an equivalent time history by monitoring the property versus time (such as strength at 3, 7, 21 and 28 days) and the internal temperature time history *T*(*t*) of the hydrating concrete. The time history can therefore be converted into *E_t_*(*t*) history at a reference temperature using the following Equation [7]:

(1) $$E_{t}\left(t\right)=\int_{t_{0}}^{t}\left[\exp\left(-\frac{E_{a}}{R}\right)\left(\frac{1}{T}-\frac{1}{T_{r}}\right)\right]dt$$

where *E_t_*(*t*) represents the equivalent time (expressed in days) at a constant temperature, (T = 20 °C in this paper); *t* and *t*_0_ represent the considered time frame; *E_a_* is the activation energy which characterizes the sensitivity of concrete hydration processes to the temperature (expressed in kJ/mol); *R* represents the universal gas constant, which is 8.314 J/(K mol); *T_r_* and *T* are, respectively, the reference (generally 293 K or 20 °C in Europe) and the observed temperature (expressed in K or °C). *E_t_*(*t*) histories are useful because at the same value of *E_t_*(*t*), a concrete property (compressive strength, by instance) is supposed to have the same value independently of the temperature history *T*(*t*) that brought to that value of *E_t_*(*t*) [13].Therefore, if the *E_t_*(*t*) curve is known for a specific concrete property, the value of that property at any time t* can be estimated by simply monitoring the internal *temperature history T*(*t*) and, using Equation (1), to determine *E_t_*(t*). The *E_t_*(*t*) histories are supposed to be directly related to *α*(*t*) histories [16], also called degree of reaction histories [18,19,20,21,22].

*E_t_*(*t*) can be calculated only if the *activation energy E_a_* is known. *E_a_* can be viewed roughly as the energy level that the reactant molecules must reach (and overcome) before a reaction can occur. For binders, the activation energy is often called “apparent activation energy” [20,21] since the cement is composed by different phases hydrating at different speeds. Therefore, the apparent activation energy is a single value representing a number of reactions. Hence, for normal concrete and for UHPC, it is critical to understand if that single value of *E_a_* is representative and can give correct predictions. UHPC often contain a mix of cement and pozzolans and hardens at high pace, and therefore a little error of the activation energy might result in large prediction errors. Methods to derive *E_a_* are always based on a simple assumption: At the same degree of reaction α*, a given physical property of concrete *P*(*α*) will assume the same value *P*(α*) = P* independently of *T*(*t*) used to reach α*. Among the concrete properties used to derive *E_a_*, and consequently both *E_t_* (*t*) and *MI*, there are the *total heat* *Q_H_*(*t*) released by the hydrating concrete (cementitious material) and the compressive strength of the concrete. Many methods to determine *E_a_* are available, some of them are standardized [1]. All these methods, such as the one presented in ASTM, C 1074-04, have their merits and proved to be of great interest, but a faster and less expensive method is of great interest for practical purposes in the field and in prefabrication plants. This paper presents a simpler and innovative method able to derive *E_a_* and, in turn, to construct the strength–*MI* curve for both normal-strength and ultra-high-performance fiber-reinforced concretes, by using a semi-adiabatic calorimetry technique. This method also allows to determine the *α*(*t*)–*E_t_*(*t*) curve, thus providing a powerful tool for quality control of hardening of cement-based materials in concrete batching plants as well as to explain the evolution of the mechanical properties of these composites [23]. The model and the tests’ procedure presented are innovative also because they make the apparatus auto-calibrating and because the model, giving a prediction with a pair of specimens, will provide over a hundred predictions with eight specimens (four runs of the apparatus). Hence, it will be easier to both spot an uneven behavior of the concrete and to statistically control the accuracy of the predictions. Finally, a further innovation of this system lies on the possibility to calculate hydration constants such as *E_a_* for the range of the rate hydration that is most adapted to the case at hand. Although many semi-adiabatic apparatuses and related models have been developed for concrete quality control, to the best of the authors’ knowledge, none presents the above-mentioned features.

### 1.1. Adiabatic Technique to Calculate the Degree of Reaction

As stated above, *E_t_* (*t*)*,* the equivalent time, is related to *α*(*t*)*,* the degree of reaction of concrete. The latter is linked to *Q_H_*(*t*)*,* the total heat released by the hydrating concrete at time *t*. *Q_H_*(*t*) can be obtained by the following Equation:

(2) $$Q_{H}\left(t\right)=m\times C_{c}\times \Delta T_{G}\left(t\right)$$

where *Q_H_*(*t*) is the total heat released by the hydrating concrete, expressed in kJ; *m* is the mass of the sample, expressed in kg; *C_c_* is the specific heat of the cementitious material, expressed in kJ/(kg K); and Δ*T_G_* is the difference between the adiabatic temperatures at time *t*_0_ and *t*, expressed in K or °C. Δ*T_G_* is measured using an adiabatic calorimeter. As *m* and *C_c_* are considered constant, *Q_H_*(*t*) is directly related with the *adiabatic temperature rise T_G_*; see Equation (2). As drawn in the Equation below, the degree of reaction has been found to be a function of the total heat released under adiabatic conditions, as follows:

(3) $$\alpha \left(t\right)=\frac{Q\left(t\right)}{Q\left(t=\infty \right)}$$

where *α*(*t*) is the degree of reaction at the time *t*, *Q*(*t*) and *Q* (*t* = *∞*) are, respectively, the total heat released at the time *t* and the total heat released in the whole reaction under adiabatic conditions, both expressed in kJ. It is therefore safe to assume that the normalized curve of the temperature rise Δ*T_G_* (*t*)*/*Δ*T_G_* (*t* = ∞) represents the evolution of *α*(*t*); see Equations (3) and (4). When the reaction is completed no further rise in temperature equals to no further heat generated (=100% degree of reaction achieved).

(4) $$\alpha \left(t\right)=\frac{Q\left(t\right)}{Q\left(t=\infty \right)}=\frac{m\times C_{c}\times \Delta T_{G}\left(t\right)}{m\times C_{c}\times \Delta T_{G}\left(t=\infty \right)}=\frac{\Delta T_{G}\left(t\right)}{\Delta T_{G}\left(t=\infty \right)}\;$$

where Δ*T_G_* (*t*) and Δ*T_G_* (*t* = ∞) are the difference between the adiabatic temperatures in the range *t*–*t*_0_ and *t_∞_*–*t*_0_, respectively. Therefore, if it is possible to measure the adiabatic temperature rise of a concrete specimen, it is possible to determine the evolution of *α*(*t*). The adiabatic *Q_H_*(*t*) can be determined by an adiabatic heat test: *T_G_* is measured on a hydrating concrete specimen for which all exchanges of heat with the surrounding environment have been somehow suppressed (i.e., there is no loss of heat).

The most common apparatuses for measuring *T_G_* of a concrete specimen are special molds with a system that measures the internal temperature of the specimen. Such a system is connected to a thermostatic device (water, electrical, etc.) installed in the external surface of the mold. The thermostatic device promptly matches the internal temperature of the concrete measured by the sensor and therefore eliminates any flux of heat to and from the specimen. Therefore, the concrete specimen hydrates in adiabatic conditions. Unfortunately, for practical purposes, the adiabatic test is not adapted due to the complexity of suppressing all heat exchanges between the specimen and the environment. For this reason, many semi-adiabatic devices have been developed to determine the adiabatic heat release of concrete.

### 1.2. Semi-Adiabatic Calorimeter (SAC) to Calculate the Adiabatic Heat Release

**Figure 1:**
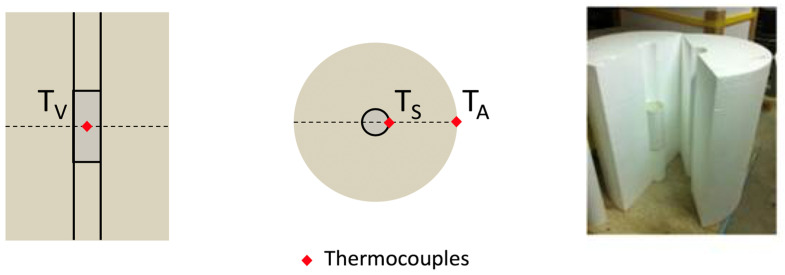
SAC apparatus and the position of the thermocouple.

If the heat exchanges are not completely suppressed, the temperature rise measured is called the semi-adiabatic temperature rise. A semi-adiabatic calorimeter (SAC) is generally a cylindrical chamber heavily insulated with the best insulating material available (see Figure 1).

The specimens of fresh concrete are placed into the chamber. Although the specimen is heavily insulated, a heat loss cannot be avoided. Nevertheless, the heat losses can be estimated, and therefore, the semi-adiabatic temperature rise can be converted into *T_G_*. In particular, Ng. et al. [24] have stated a simple equation to compensate the temperature loss, based on temperature (gradient) measurement:

(5) $$T_{G}\left(t\right)=\left(T_{V}-T_{P}\right)+\lambda \int_{0}^{t}\left(T_{S}-T_{A}\right)dt$$

where *T_G_* is the adiabatic temperature rise of concrete sample at the time *t*; *T_V_* is the volumetric mean temperature of concrete sample inside a SAC; *T_P_* is the placing temperature of concrete sample; *λ* is the heat-loss compensation factor which is observed in semi-adiabatic apparatus; *T_S_* is the mean temperature of the concrete sample surface; *T_A_* is the SAC surface temperature close to the ambient. In Equation (5), all temperatures can be measured with simple thermocouples, while the heat-loss compensation factor *λ* must be calculated. *λ* can be assumed, measured, or estimated experimentally.

Among the most used methods to determine the adiabatic *Q_H_*(*t*) of concrete specimens, there are the QAB and the *Langavat* calorimeters [5]. Both devices are normalized according to [25] and require calibration to determine both *λ* and the heat capacity of the calorimeters. This is required because the heat-loss compensation is made by estimating the heat absorbed by the insulation. These two devices were not designed to calculate *E_a_*. However, the QAB calorimeter can be easily modified and used for this purpose, as is proposed in this paper. SAC is both a simplified and a modified version of the QAB, whose reliability has been confirmed by several works [26,27,28,29,30,31,32,33].

### 1.3. Standard and Non-Standard Methods to Determine the Heat-Loss Factor λ

**Figure 2:**
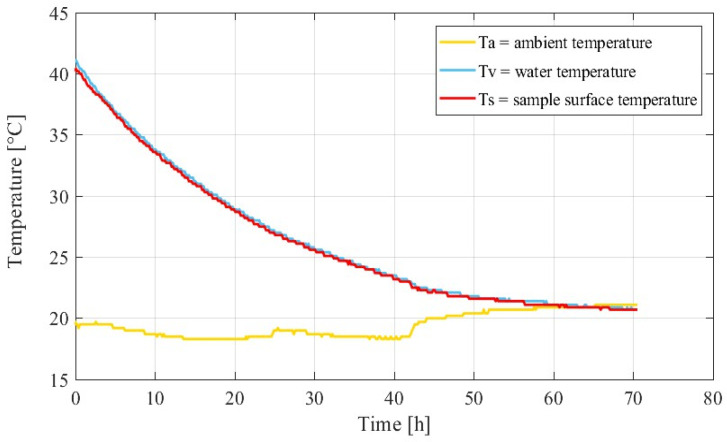
λ determined by authors with Nordtest (www.nordtest.org, accessed on 19 December 2021) [34] and standard methods ASTM [1]: calibration with ASTM (hot water) temperature history.

The value of *λ* could be obtained by simply calibrating the apparatus using hot water. In particular, a specimen of water at high (and known) temperature is inserted into the chamber of a SAC (see Figure 2). Temperatures are measured, and the heat flux is estimated, hence *λ* turns out to be

(6) λ(t)=∂TV∂t(Ts−TA)

where ∂*T_V_/∂t* is the gradient of the volumetric mean temperature of a concrete sample along the time. This is the principle of the well-known *Nordtest* method [34]. A more practical approach to determine *λ* has been proposed by Ng et al. [24] who apply Equation (2) by using temperature values measured while a concrete specimen hydrates inside the SAC (see Figure 2). The method of Ng et al. has a distinct advantage: it permits to self-calibrate the device while a concrete specimen is being tested. In addition, this method allows obtaining a new estimation of *λ* every time a test is run. Given that all parameters of Equation (5) can be easily determined, it is therefore established that a semi-adiabatic temperature rise can be transformed in *T_G_*.

### 1.4. Determination of the Activation Energy by Using Adiabatic Heat Curves

In order to derive *E_a_*, Equations (1) and (2) are used: two identical specimens of hydrating concrete are supposed to have released the same quantity of heat per unit of mass, when the reaction is completed (*α* = 100%, see Equation (3)) and also at whatever degree of reaction *α*. If the objective is to determine *E_a_* by using adiabatic heat curves, two conditions have to be satisfied:

- It must be possible to measure or derive the adiabatic heat of hydration of two specimens; see Section 1;
- The two specimens must not have the same initial temperature and temperature rise.

If both conditions listed above are satisfied, the semi-adiabatic temperature rise of the two specimens can be measured and therefore converted into *T_G_*. *T_G_* values derived for both specimens are finally normalized to the specimen weight. The normalized adiabatic temperature rises Δ*T_G_* (*t*)*/*Δ*T_G_* (*t* = ∞) represent the evolution of *α*(*t*) according to Equations (3) and (4). *Speed* and *superposition* methods [34] can be used to decouple *E_a_*. A variant of both methods is the *equivalency points* method [22] that allows determining a value of *E_a_* for every point of equal *α*(*t*) of two specimens. The equivalency point method determines *E_a_* by simply calculating for two different temperature histories *T*_1_(*t*) and *T*_2_(*t*) of two different specimens the values of *Et*_1_(*t_1_*) and *Et*_2_(*t*_2_) up to a common degree of reaction α*. By equating the two values of *E_t_*, the activation energy is determined.

## 2. Materials and Methods

It is evident that the fastest procedure to obtain *E_a_* is to construct two or more SACs working in parallel on concrete specimens sampled from the same batch. The two specimens must have a different initial temperature to ensure they will not have the same *T*(*t*)*,* in agreement with Section 1.4. As stated previously, a SAC is a chamber surrounded by a heavy layer of insulation. In the present research, a chamber was cut inside a cylinder of EPS, which is a commonly used insulation material for buildings’ walls. The industrial grade of the EPS used permitted to both have all cylinders cut from the same element and ensure constant characteristics of the materials. The cylinders of SAC were equipped with standard K-type temperature sensors with ±1 degree of incertitude in a temperature range of 0–400 °C. The temperature probes were placed at several points in the SAC, as shown in Figure 1. Temperature probes were connected to a programmable data reader/logger. Two thin-walled plastic cylinders were used as molds for the fresh concrete and perfectly fitted the central hole in the EPS cylinders; see Figure 1.

Heat loss compensation of the semi-adiabatic temperature rise can be achieved only if *λ* of Equation (6) is known. Thanks to the test procedure presented by Ng et al. [24] (see Equation (5)), the SAC can be considered a self-calibrating device. In fact, the lambda value could be found by calibrating the apparatus with hot water, but the procedure of Ng et al. permits to find two *λ* values each time a test is run, making the apparatus auto-calibrating. In order to verify the suitability of the self-calibration procedure of the SAC, a calibration test similar to the Nordtest method was also performed. This is important because *λ* value tends to slightly vary with the temperature of the specimen. Hot water was inserted in the SAC chamber, and temperatures were monitored at the location shown in Figure 1. Figure 2 shows the temperature measurement on the different thermocouples during 70 h. After 60 h, the equilibrium between ambient and water temperature was reached.

Calculated *λ* values were respectively 0.0422 (hot water) and 0.0475 (concrete). The calibration tests therefore agree with the principle presented by Ng et al. [24], as a single *λ* value can be found. Furthermore, a new *λ* value is obtained each time a test is run. Consequently, it is easy to control if the values obtained are reasonable and stable at the typical temperatures recorded. It should be noted that a *λ* value calculated by Ng et al. [24] is not the value of *λ* that characterizes building insulation materials.

In order to prove the suitability of the proposed method for commercial concretes of different mixture composition, two concretes were investigated (see Table 1 and Table 2): a commercial UHPC with a compressive strength of about 151 MPa [35] and an ordinary concrete classified as C30/37 (labeled hereinafter OC-C30/37), according to the SN EN 206 standard. UHPC are used to build structures [36], as well as to manufacture products such as tabletops, facades and chairs. Codes as SETRA and SIA provide guidelines to both characterize the UHPC and design UHPC structures [37,38]. UHPC is a material that, after a long dormant period (often shortened using accelerators) shows a vivid reaction, reaching high temperatures. The OC-C30/37 is a multipurpose structural concrete, adapted to build most structures and used in large quantities. Two tests of the OC-C30/37 specimens were made using batches made in controlled conditions, taking care that the w/c ratio was respected, as requested in certified and modern ready-mix concrete plants. The third couple of OC-C30/37 specimens were taken from a batch made in conditions similar to the ones commonly found in little construction fields operating a small, in-field plant. In particular, the aggregate was stockpiled in the open air, by assuming that the aggregate humidity remained constant after a first control. All batching operations were executed as carefully as possible given the boundary conditions.

Mixing was performed using a high shear rotating pan mixer. This mixer was chosen because it is close to the efficiency of the industrial mixers used for the production in large scale of the concrete materials similar to those here investigated; see Table 1 and Table 2. Only for a batch of OC-C30/37, a drum mixer was used. Tests were performed on both UHPC and OC-C30/37 series. Each series is composed of 3 to 4 specimens. Some isothermal tests were held using an isothermal calorimeter [39,40,41,42], and mechanical tests were carried out using a universal compression machine.

## 3. Results

### 3.1. UHPC Series

To maintain the repeatability of the SAC test, three UHPC series were tested. Data collected were used to determine three different *E_a_* values of UHPC. First, the SAC tests were conducted on specimens at different initial temperatures, as proposed in Section 1. Furthermore, the obtained semi-adiabatic *Q_H, SAC_*(*t*) curves were compensated by using Equation (5) to obtain *Q_H_*(*t*) of each series as a function of the time. Then Equation (4) was used to determine *α*(*t*) for each specimen. Finally, using the principle of equivalency points explained in Section 2 and in [22], *E_a_* was determined for each couple of series (S1–S2, S1–S3, S2–S3). *E_a_* values obtained for the three series are listed in Table 3. These values are obtained as an average of the values obtained for each couple of series using the concept of the equivalency points for different values of *α*(*t*).

All these calculations were made using a commercial computational code. Data reduction included also a curve smoothing based on the Savitzky–Golay algorithm. Two types of incertitude were encountered in the determination of *E_a_*. The first one was related to the use of the smoothing algorithm Savitzy–Golay. The second one was due to the fact that often *Q_H, SAC_* (*t*) curves are both overlapping and crossing, thus making more complex the determination of the correct *E_a_* value. Both sources of uncertainties are easily eliminated by simply repeating regularly the SAC tests in order to have a significant number of values. Using a basic regression technique, the incoherent data can be filtered. The signature provided by *Q_H_*(*t*) curves and the temperature measurement are also useful to signal the occurrence of a sudden change of quality of commercial UHPC. In fact, in order to meet the designer’s requirements, e.g., self-compacting UHPC, thixotropic UHPC and projected UHPC, the producers adapt the UHPC mixture (compounds and plasticizers). In a previous work [43], a commercial UHPC was tested in order to fabricate a series of beams for the roof of a large restaurant. In the early phases of the UHPC beam production, the temperature measurement of the maturometers signaled a setting time of the fresh mixture up to 48 h. This phenomenon, which was due to a change of the polymers blend used to fabricate the superplasticizer, was corrected by the use of an accelerator. *E_a_* and *Q_H_*(*t*) curves were not affected by this phenomenon.

Another incertitude concerns the determination of the *initial point t*_0_, i.e., the moment at which the hydration begins. Controversial opinions about methods for the identification of *t*_0_ as well as the determination of the setting time are found in the literature [21,22].

The graphical method proposed by Viviani et al. [22] was used in this work, proving to be efficient.

**Figure 3:**
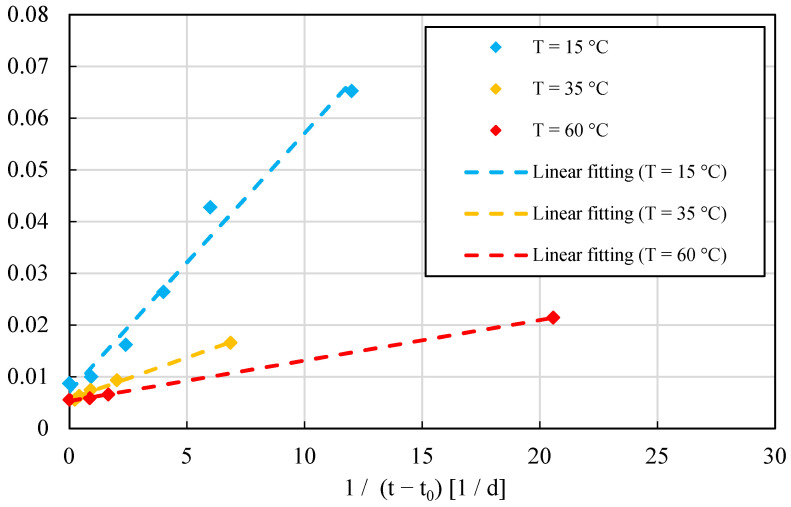
Determination of *E_a_* for UHPC series according to standard test (ASTM 1074). Each point corresponds to the mean value among 3 to 4 observations.

In order to confirm the results provided from SAC tests, a standard test (ASTM 1074) was performed to determine the value of *E_a_*; see Figure 3. In such a figure, dashed lines denote linear fits of the experimental data (for advanced fitting procedures see, e.g., [44,45]). The standard procedure recorded a *E_a_* value of 30.614 kJ/mol, in agreement with data found in the literature [19,20]. This comparison confirms the reliability of the method presented in this paper which results faster and less expensive than the standard test.

**Figure 4:**
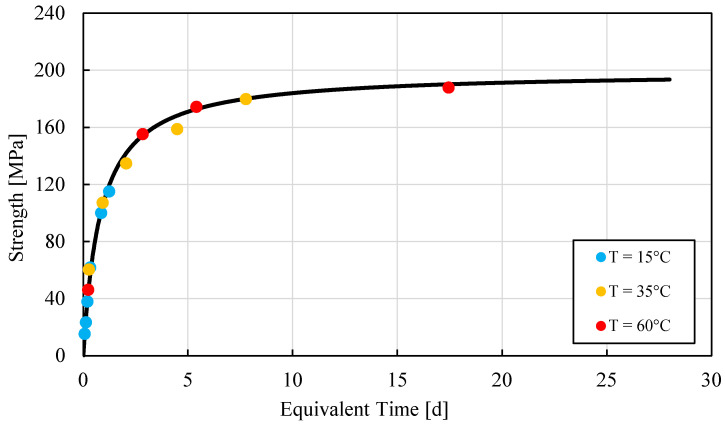
Strength−Equivalent Time curve for UHPC specimens. Each point corresponds to the mean value among 3 to 4 observations.

Finally, a strength–*MI* curve was developed to determine the strength of the UHPC at any time. Most points along the strength–*MI* curve were determined for the early age of UHPC. A single value of *E_a_* proved to be sufficient for the 28-day strength. The strength–*MI* curve was developed specifically for a precaster whose main need was to cast a relevant number of post tensioned beams in less than 20 h. The standard test mentioned above also provided and *E_a_* value. The obtained strength*–E_t_*(*t*) curves were confirmed by plotting independent strength test values in Figure 4.

**Figure 5:**
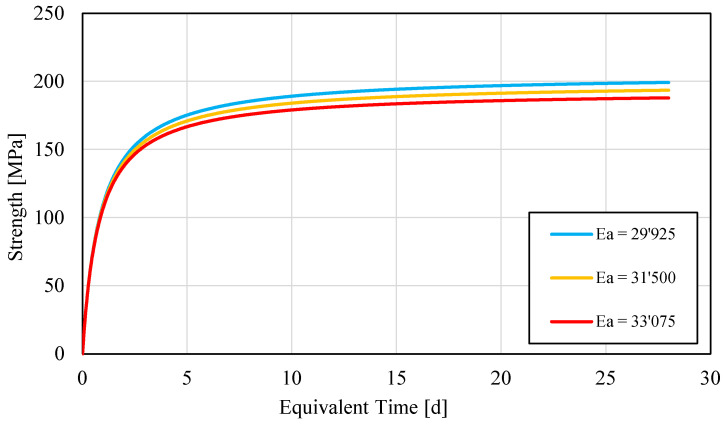
Sensitivity analysis of UHPC series: Strength–Equivalent Time test values for different *E_a_* values.

One of the main issues concerning the maturity methods used in the field is to determine if the strength–*E_t_*(*t*) curve is representative of the hydration process of the cement-based material, for the ambient temperature and time of strength desired [20,21]. In order to minimize such a risk, it is reasonable to prepare an adequate number of specimens and compare their actual strength with the predicted strength–*E_t_*(*t*). A sensitivity analysis allows uncovering the effect of the errors on the predictions of strength–*E_t_*(*t*) curves, which is caused by the determination of different *E_a_* values, as already discussed above. A sensitivity analysis was therefore performed: the *E_a_* value was changed by a ±10% value, and the strengths at 1, 3 and 28 days were recalculated. The maximum difference between the original predicted value and the values obtained by altering *E_a_* value were lower than 6.5%, 5% and 3% for 1, 3 and 28 days of *E_t_*(*t*), respectively (see Figure 5).

**Figure 6:**
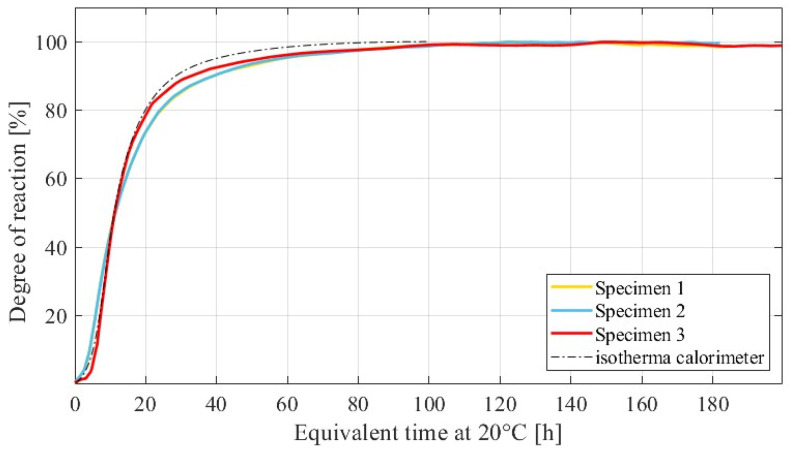
Comparison between Degree of reaction−Equivalent Time curves from SAC and isothermal calorimetric tests on UHPC specimens.

One of the interesting points in the use of the SAC method is that *Q_H_*(*t*) curves are available, and thus, it is possible to calculate an approximation of the evolution of the *α*(*t*) of each specimen as a function of the *E_t_*(*t*); see Equation (3). Figure 6 presents the *α*(*t*) curves calculated via a SAC test, which are expressed as a function of *E_t_*(*t*) at 20 °C for UHPC specimens. Different batches of cement-based materials can lead to a small scatter between *α*(*t*) curves, as can be observed in Figure 6. However, the reliability of such curves was confirmed by superposing the latter to the curve obtained by using an isothermal calorimeter. The match between the curves is good, even though the compounds of the specimen monitored in the isothermal calorimetry could not contain a representative percentage of the largest aggregates present in the UHPC nor of the steel fibers. This drawback is due to the limited size of the standard vessel of isothermal calorimeters, whose analyses cannot be representative for concrete materials.

### 3.2. OC-C30/37 Series

Three couples of OC-C30/37 series presented in Table 2 were tested using the SAC method. The specimens were cast in laboratory using the mix design used by a ready-mix plant and with a close control of the batching parameters (series of the couple 1 and 3). The batch of the couple 3 was made using the same mix design, but the mixing was performed by a drum mixer, and aggregates were stockpiled in open air; see Section 3. The software and method used to both compute the experimental data and identify *t*_0_ of hydration were the same presented in Section 3.1. An *E_a_* value was determined for each couple of series; see Table 4. These values were obtained by averaging the values derived for each specimen, by using the same concept presented in Section 3.1, i.e., the equivalency points for different values of *α*(*t*).

**Figure 7:**
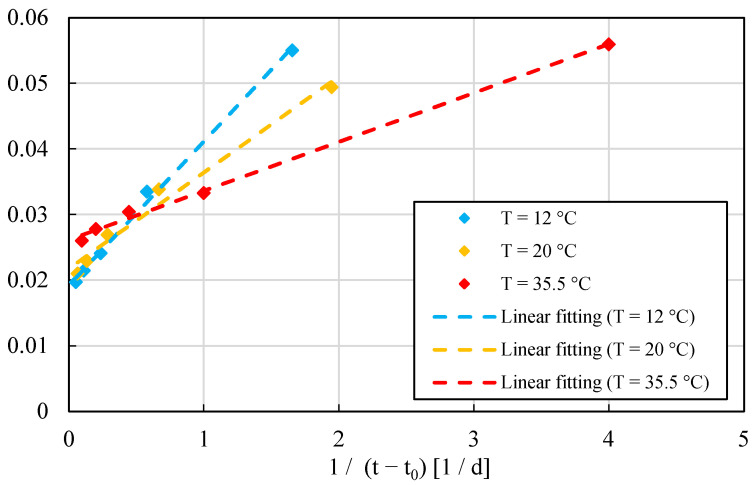
Determination of *E_a_* for OC-C30/37 series according to standard test (ASTM 1074). Each point corresponds to the mean value among 3 to 4 observations.

*E_a_* value was verified also by performing the standard test ASTM 1074, as presented in Figure 7. Standard test results provided *E_a_* value of 42.916 kJ/mol, in agreement with data found in the literature. This comparison confirms the accuracy provided by the method presented in this paper for any concrete class (normal and ultra-high). A strength–*MI* curve was developed to determine the strength of OC-C30/37. A single value of *E_a_* proved to be sufficient for the 28-day strength.

**Figure 8:**
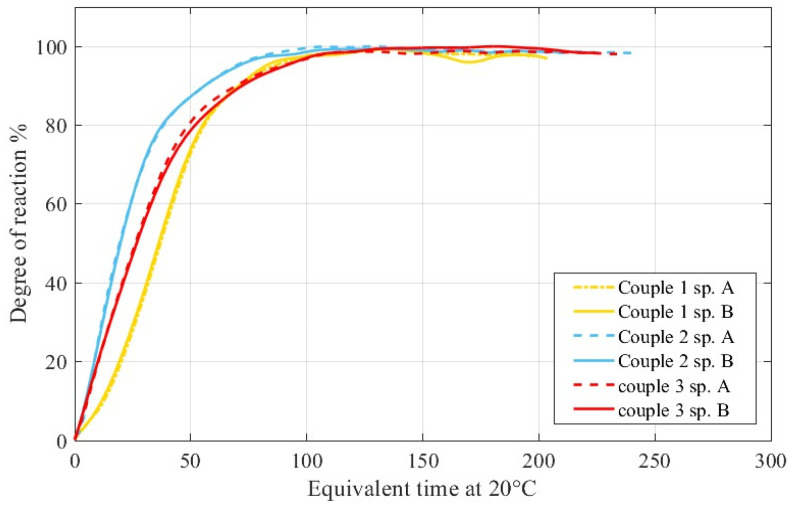
Comparison between Degree of reaction−Equivalent Time curves: OC-C37 series monitored in SAC. Series of the couple “2” where manufactured differently.

The evolution of *α*(*t*) for OC-C30/37 series as a function of the equivalent-time at 20 °C is reported in Figure 8. The direct comparison with the isothermal calorimetry was not possible in this case since the ordinary concrete contains large aggregates not compatible with a standard vessel of the isothermal calorimeter. Figure 8 confirms that the batches made with controlled conditions similar to an industrial plant are close and match well with a small error. The curve of the second couple of series proved instead to be close but yet not matching. This is a clear indication of the importance and correctness of the dispositions of the norm EN 206 for ensuring a constant quality of the structural concrete, especially with regards to the control of the humidity of the aggregates and to the interdiction of using “all-in-one” aggregates for certified structural concrete [46].

OC-C37 proved to be a good structural concrete for general purposes, but it is not an ideal candidate for the type of field that relies heavily on Maturometry to speed up the construction planning. In fact, Figure 7 shows a rather pronounced temperature sensitivity when the concrete is cured at 35 °C. This phenomenon might affect the predictions when the temperature rises.

## 4. Discussion

**Figure 9:**
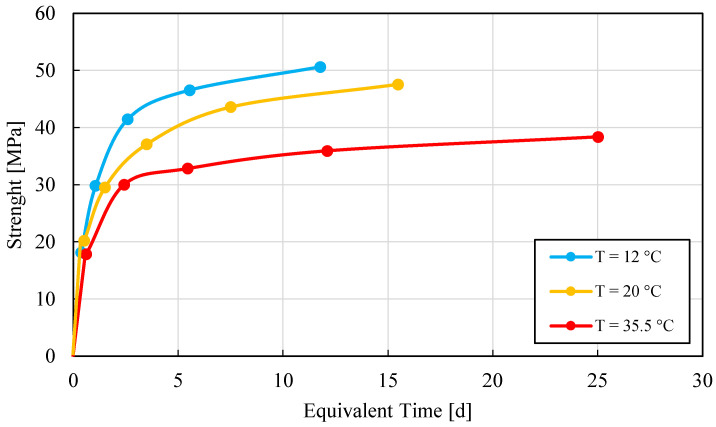
Strength–Equivalent Time test value at three different temperatures. Each point corresponds to the mean value among 3 to 4 observations.

The proposed method shows a great potential. It can be used to quickly derive the *E_a_* of representative concrete specimens, from normal-strength to ultra-high-performance fiber-reinforced concretes. Once *E_a_* value is determined, an accurate plot about the evolution of the rate of reaction *α*(*t*) and therefore the strength–*MI* curve is computed. Most of the theoretical background on which the SAC test is based has been already validated by other studies and practitioners [13,17,18,19,20,21,28,29,30]. The great advantage of using calorimetric techniques such as the SAC, especially if adiabatic curves are derivable, is that they constitute an easy and powerful quality control method, able to detect any variation of w/c ratio, change of quality or type of cement, compatibility problems with a new plasticizer and unexpected changes in the mix proportions. In addition, SAC test is self-calibrating (see Section 2), and it permits to statistically improve the quality of the predictions of *E_a_* values, as the number of specimens tested increases. There are no particular risks using this technique as far as the users have an adequate knowledge of concreting and concrete. Some risks arise when the predictions of the strength–*MI* curves are provided on the basis of hardened specimens monitored under similar temperature profiles. In fact, cement-based materials showing a strong crossover effect [47], might underperform if hydrating at high temperatures. The crossover refers to a lower final strength of a concrete curing at high temperatures during the early hydration age compared with the same concrete curing at lower early age temperatures. This was the case of the OC-C30/37 series, which showed a marked crossover effect when the specimens were conditioned at high temperature at the beginning of the hydration; see Figure 9. In this case, as for the UHPC series, the curves were constructed and validated using specimens hardened under different hydrating temperature conditions, and hence, the crossover effect was easily spotted; see Figure 7 and Figure 9. The accumulation of the errors connected both with smoothing techniques and the propagation of errors is always an issue in all measurement techniques. The Savitzky–Golay model is a well-known and efficient algorithm, but the driving parameters must be carefully evaluated to avoid that the original curve be excessively modified. The propagation of measurement errors, such as the uncertainties related to the temperature measurement, might be the primary cause of scattered values of both *E_a_* and evolution of *α*(*t*).

Another important point for this technique is that the specimens should be of equal mass; otherwise, the curves of the temperature might cross each other making the data collected useless (most of the times). Hence, the specimens’ holders must be completely filled, and their weight controlled. Finally, the degree of hydration–Et curves (see Figure 6) constitutes a powerful quality control tool. A variation of mix, mix proportion or quality and any other issue for a specific concrete batch can be easily spotted if these curves suddenly change for a specimen. Beside the variation of the quality of the concrete, a variation of these curves will be a red flag for the use of the strength predictions.

## 5. Conclusions

This paper presents a fast and inexpensive method to determine the strength–Et evolution of both normal-strength and UHPC concretes. Results provided by the proposed method were compared with those obtained by standard tests, showing great agreement. Based on the results of this research, the following conclusions can be drawn:

- The SAC test is an accurate, inexpensive, fast and self-calibrating method to calculate the activation energy of cement-based materials;
- The quality of predictions of strength–maturity curves provided by the proposed method can be constantly improved as the specimens’ population increases;
- The SAC test is a good quality control tool since it can detect any variation of w/c ratio, change of quality or type of cement, compatibility problems with a new plasticizer and unexpected change of the mix proportions;
- This fast and inexpensive method to control quality of any concrete class is limited by the fact that values for the activation energy are obtained under specific temperature conditions and might not accurately represent the value of *E_a_* under a much different temperature. A method which can overcome this limitation is the resistivity model based on Winner bounds, even though available data show that it is still not suitable for concrete due to the poor electrode contact and non-uniformity caused by the presence of coarse aggregate.

## Figures and Tables

**Table 1:** Components in UHPC.

Component	Kg/m^3^
Premix ^1^ (cement, aggregate 0–6, silica fume)	2296
Water	180
Superplasticizer ^1^	40
Set and hardening accelerator ^1^	20
Steel fibers ^2^ (20/0.3 mm)	195

^1^ Producers did not provide the specific commercial mixture composition. What is known is that premix contains aggerate composed of calcined bauxite residues. ^2^ Specimens were investigated without steel fibers. Further details about steel fibers are reported in [35].

**Table 2:** Component in OC-C30/37.

Component	Kg/m^3^
Cement CEM II/A LL 425N	340
Water	164
Plasticizer	0.015
Rheology modifier	0.005
Aggregate (0–32) SSD	1945

**Table 3:** *E_a_* for UHPC series.

Specimen Couple	Activation Energy *E_a_*
S1–S2	31,161
S1–S3	27,437
S2–S3	32,772

**Table 4:** Activation energy *E_a_* for OC-C30/37.

Series Couple	Activation Energy *E_a_*
Couple 1	40,441
Couple 2	44,595
Couple 3	37,485

## Data Availability

Data sharing not applicable.
